# Statistical process control of mortality series in the Australian and New Zealand Intensive Care Society (ANZICS) adult patient database: implications of the data generating process

**DOI:** 10.1186/1471-2288-13-66

**Published:** 2013-05-24

**Authors:** John L Moran, Patricia J Solomon

**Affiliations:** 1 Department of Intensive Care Medicine, The Queen Elizabeth Hospital, 28 Woodville Road, Woodville, SA 5011, Australia; 2 School of Mathematical Sciences, University of Adelaide, Adelaide, SA 5000, Australia

**Keywords:** Statistical process control, Time series, Autocorrelation, Seasonality, Volatility, Exponentially weighted moving average smoothing, Autoregressive moving average models, GARCH models

## Abstract

**Background:**

Statistical process control (SPC), an industrial sphere initiative, has recently been applied in health care and public health surveillance. SPC methods assume independent observations and process autocorrelation has been associated with increase in false alarm frequency.

**Methods:**

Monthly mean raw mortality (at hospital discharge) time series, 1995–2009, at the individual Intensive Care unit (ICU) level, were generated from the Australia and New Zealand Intensive Care Society adult patient database. Evidence for series (i) autocorrelation and seasonality was demonstrated using (partial)-autocorrelation ((P)ACF) function displays and classical series decomposition and (ii) “in-control” status was sought using risk-adjusted (RA) exponentially weighted moving average (EWMA) control limits (3 sigma). Risk adjustment was achieved using a random coefficient (intercept as ICU site and slope as APACHE III score) logistic regression model, generating an expected mortality series. Application of time-series to an exemplar complete ICU series (1995-(end)2009) was via Box-Jenkins methodology: autoregressive moving average (ARMA) and (G)ARCH ((Generalised) Autoregressive Conditional Heteroscedasticity) models, the latter addressing volatility of the series variance.

**Results:**

The overall data set, 1995-2009, consisted of 491324 records from 137 ICU sites; average raw mortality was 14.07%; average(SD) raw and expected mortalities ranged from 0.012(0.113) and 0.013(0.045) to 0.296(0.457) and 0.278(0.247) respectively. For the raw mortality series: 71 sites had continuous data for assessment up to or beyond lag_40_ and 35% had autocorrelation through to lag_40_; and of 36 sites with continuous data for ≥ 72 months, all demonstrated marked seasonality. Similar numbers and percentages were seen with the expected series. Out-of-control signalling was evident for the raw mortality series with respect to RA-EWMA control limits; a seasonal ARMA model, with GARCH effects, displayed white-noise residuals which were in-control with respect to EWMA control limits and one-step prediction error limits (3SE). The expected series was modelled with a multiplicative seasonal autoregressive model.

**Conclusions:**

The data generating process of monthly raw mortality series at the ICU level displayed autocorrelation, seasonality and volatility. False-positive signalling of the raw mortality series was evident with respect to RA-EWMA control limits. A time series approach using residual control charts resolved these issues.

## Background

Statistical process control (SPC), deriving from Shewart’s work in 1920-30 and in the 1950’s with Deming’s refinements [1], has been more recently applied in health care and public health surveillance [2], generating considerable interest in the general [3-5] and specialist medical literature [6-10]; and has been subject to a detailed exposition from a “quality-in-medicine” perspective [11]. Important statistical principles underlying SPC or control-chart methodology are those of the monitored process being “in control” and subject to the independence of observations [12]. The presence and impact (possible increase in frequency of false alarms) of process autocorrelation in industrial/engineering series have long been documented [13-16]. Somewhat surprisingly, little formal attention has been directed to this problem in the bio-medical literature [17,18], one review suggesting that there was “…limited advice on how to manage [autocorrelation]…” [5].

We have previously drawn attention to the data-generating mechanisms of overall monthly mortality series, at the aggregate level, from a bi-national intensive-care (ICU) database, where persistent autocorrelation (to lag_24_) was evident in a seasonal ARIMA (auto-regressive integrated moving average) model of the mortality series [19]. We now extend this study to further characterise the data generating process of mortality series at the individual ICU level and the impact of autocorrelation upon (i) mortality monitoring using EWMA (exponentially weighted moving average) control charts and (ii) time-series modelling of the data process using residual control charts.

## Methods

As previously described [19,20], the ANZICS (Australian and New Zealand Intensive Care Society) adult patient database [21] was utilised to define an appropriate patient set, 1995-(end)2009. Physiological variables collected in accordance with the requirements of the APACHE III algorithm [22,23] were the worst in the first 24 hours after ICU (intensive care unit) admission, and all first ICU admissions to a particular hospital for the period 1995-2009 were selected. Records were used only when all three components of the Glasgow Coma Score (GCS) were provided; records for which all physiologic variables were missing were excluded, and for the remaining records, missing variables were replaced with the normal range and weighted accordingly. The mortality endpoint was at hospital discharge. Exclusions: unknown hospital outcome; patients with an ICU length of stay ≤ 4 hours, and patients aged < 16 years of age. Access to the data was granted by the ANZICS Database Management Committee in accordance with standing protocols; local hospital (The Queen Elizabeth Hospital) Ethics of Research Committee approval was waived.

### Statistical analysis

(i) Monthly raw (risk-unadjusted) and risk-adjusted (RA) mortality time series at the individual ICU were generated. Risk adjustment was undertaken, generating the “expected” series, using a random coefficient logistic model (intercept as ICU site and “slope” as (centred) APACHE III score; unstructured covariance using adaptive quadrature, estimated via the Stata™ module “xtmelogit” [24]), as previously described in detail [20], and extended to both ventilated and non-ventilated patients. No formal adjustment for potential seasonality (trigonometric seasonality using sine/cosine functions or monthly dummy variables) was undertaken. Individual ICUs were allocated an identifier based upon a random number sequence.

(ii) Graphical inspection of the mortality series and formal testing of normality to confirm that the “…distributions of… (observed) and… (predicted) [series] … were sufficiently similar and are robustly normal and symmetrical…” [25]. Classical seasonal decomposition [26] was undertaken using the “decompose” module in R statistical software (Version 15.2 [27]). Autocorrelation plots (scatterplot grid of series versus lagged values) were performed via the R user-written module “lag1.plot” [28].

(iii) Generation of EWMA charts with confidence limits.

a. assuming iid (independent and identically distributed) observations, the EWMA statistic (*z*_*i*_) is defined as: *λx*_*i*_ + (1 − *λ*)*z*_*i*−1_ and the variance (*σ*^2^) as $\sigma_{z_{i}}^{2}=\sigma_{x}{}^{2}\left(\frac{\lambda }{1-\lambda }\right)\left[1-{\left(1-\lambda \right)}^{2i}\right]$, where 0 <*λ* ≤ 1 is a constant (smoothing parameter) [29].

a. For the variance of (non-stationary) auto-correlated series, we followed Montgomery & Mastrangelo [15]: division of the sum of squared (prediction) errors for optimal λ by *n*; leading to the plotting of a moving centre-line EWMA control chart [12].

a. Default values (“optimal”) in Stata™ statistical software for λ were chosen to minimize the in-sample sum-of-squares forecast errors [30], a method also recommended by Montgomery and Mastrangelo [15]; albeit small values of λ may inhibit the detection of large sudden process shifts; the “inertia” phenomenon [31].

a. Average run length (ARL): that is, the average number of “points”, when the data-generating process is in fact in-control, plotted before out-of-control is declared (ARL_0_). For instance, with iid observations and a Shewhart control-chart with three sigma limits, ARL_0_= 1/*p*=1/0.0027=370 (where p is the probability that any point exceeds the control limits [32,33], when the data-generating process is in fact in-control). Under the iid assumption, for various mortality series and values of λ, scenario based increments of the (mean of the) underlying series were computed using Statgraphics® Centurion XVI statistical software [34].

a. Using conventional SPC methods, EWMA control limits (at 3 sigma) were applied to the raw mortality series using the expected series as reference process; that is, RA control limits were generated.

(iv) Establishment of time-series models at the individual ICU level was based upon classic Box-Jenkins methodology (autoregressive moving average (ARMA) models) with investigation of (G)ARCH ((Generalised) Autoregressive Conditional Heteroscedasticity) effects [35,36], as previously described [19].

a. A stationary time series {*x*_*t*_; *t* = 0, ± 1, ± 2, …} has an autoregressive moving average (ARMA(*p*,*q*)) structure: *x*_*t*_ = *ϕ*_1_*x*_*t* − 1_ + … *ϕ*_*p*_*x*_*t* − *p*_ + *ω*_*t*_ + *θ*_1_*ω*_*t* − 1_ + … *θ*_*q*_*ω*_*t* − *q*_ where *ϕ*_1_, *ϕ*_2_, …, *ϕ*_*p*_ are the “autoregressive” (AR) coefficients relating the value of *x* at time *t* to its past *p* values, and *θ*_1_, *θ*_2_, …, *θ*_*q*_ are the “moving average” (MA) coefficients, relating the current “white-noise”,*ω*_*t*_, to its past *q* values and $\omega_{t}\sim N\left(0,\sigma_{\omega }^{2}\right)$. If *x*_*t*_ has a non-zero mean (*μ*), then a constant *α* = *μ*(1 − *ϕ*_1_ − … − *ϕ*_*p*_) is introduced into the structure. An integrated series accumulates (some) past effects and is therefore non-stationary. A series is integrated, say, of order 1 (*I*(1)) if the changes (or differences: *Δx*_*t*_ = *x*_*t*_ − *x*_*t* − 1_) of the series generate stationarity (*I*(0)), leading to the expanded ARIMA model (ARIMA(*p,d,q*)), where *d* is the degree of differencing [37]. This being said, careful attention was directed to the question of trend versus difference stationarity [38], especially in medical series where, as opposed to stochastic random walks, “deterministic” trends may be present. [39,40].

a. Model diagnostics: the use of auto- (ACF) and partial-autocorrelation (PACF) function displays, testing for the presence of a unit-root (ADF (augmented Dickey-Fuller) and DF-GLS (modified Dickey–Fuller *t* test) tests [30] and variants), residual white-noise (Bartlett’s periodogram-based- and Portmanteau (Q)-test) and seasonality were undertaken after Shumway & Stoffer [41] and as previously described [19].

a. Volatility of the (squared) residuals (*ϵ*) of the mean equation (conditional heteroscedasticity [42]) was checked using the PAC of the squared residuals and the user-written Stata™ “armadiag” module [43]; that is, ARCH and GARCH effects ((Generalised) Autoregressive Conditional Heteroscedasticity of the error variance process). For an ARCH model, the mean equation is *y*_*t*_ = *x*_*t*_*β* + *ϵ*_*t*_ and the variance equation $\sigma_{t}^{2}=\gamma_{0}+\gamma_{1}\epsilon_{t-1}^{2}+\gamma_{2}\epsilon_{t-2}^{2}+\ldots$, where $\epsilon_{t}\sim N\left(0,\sigma_{t}^{2}\right)$, $\epsilon_{t}^{2}$ are the squared residuals (innovations) and *γ*_*i*_ are the ARCH parameters; the conditional variance is thus modelled as an AR process. A GARCH(*m*,*k*) model includes lagged values of the conditional variance

$\left(\sigma_{t}^{2}=\gamma_{0}+\gamma_{1}\epsilon_{t-1}^{2}+\gamma_{2}\epsilon_{t-2}^{2}+\ldots +\gamma_{m}\epsilon_{t-m}^{2}+\delta_{1}\sigma_{t-1}^{2}+\delta_{2}\sigma_{t-2}^{2}+\ldots +\delta_{k}\sigma_{t-k}^{2}\right)$, where *δ*_*i*_ are the GARCH parameters (an ARMA process) [19,44]. Exploration of different error term distributions (normal, *t* and generalised error) was also undertaken [30].

a. Under the conditions of an appropriately specified time-series model, the behaviour of the residuals was investigated, after Alwan and Roberts [45], on the basis that a shift in the mean of a time series is transmitted to the residuals [46].

b. As residuals are assumed to be independent (white-noise: a sequence of iid random variables with finite mean and variance, all ACFs being [close to] zero [47]), standard control chart methods were used to generate residual-EWMA charts [33]. Thus, determination of the residual-EWMA smoothing parameter (λ) was based upon methods for independent observations.

b. Control limits were also determined using standard errors (3×) of the one-step-ahead forecasts [45].

a. Model selection was guided by penalized information criteria (Akaike (AIC) and Bayesian (BIC) information criteria) [48].

a. Formal exegesis proceeded using a single exemplar complete ICU series (1995-(end)2009).

(v) Graphical displays: line-graphs of series were produced for appropriate illustration of relevant stages of analysis

a. Line graph(s) of the raw series were produced with 3*SE control limits of the expected series.

a. EWMA control limits (including residual control charts) were generated using default values of “optimal exponential coefficient” in Stata™ statistical software [49].

a. Values of λ for scenario based increments (say, 5% or 10%) of target mean were calculated using the SPC module of Statgraphics® statistical software [34] and appropriate 3*SE control limits of the expected series as in (a) above or EWMA line graphs were produced as in (b) above.

## Results

The overall data set, 1995-2009, consisted of 491324 records from 137 ICU sites; mean (hospital) mortality was 14.07%. The random coefficient logistic regression model (Hosmer-Lemehsow statistic 62.97, ROC area under the curve 0.89) generated an overall predicted mortality probability of 0.1407 (SD 0.0202, range 0.00004-0.993). Over the 137 sites mean raw and expected (RA-) mortalities ranged from 0.012(0.113) and 0.013(0.045) to 0.296(0.457) and 0.278(0.247) respectively.

Of the raw mortality series from the 137 ICUs, 71 had continuous monthly data (excluding missing values or zero monthly mortality) for assessment up to or beyond lag_40_. For 25 of these series (35%), there was a significant Q test (null hypothesis being that the series is white noise) and autocorrelation through to lag_40_. Thirty six had continuous monthly data (excluding missing values) for ≥ 72 months; all series demonstrated marked seasonality and 30 demonstrated an obvious trend decline in mortality. Of the expected mortality series, 72 had appropriately assessable data to lag_40_ and in 46 (64%) there was a significant Q test and autocorrelation through to lag_40_. Similarly, in the same 36 series with continuous (raw) monthly data for ≥ 72 months, all expected mortality series demonstrated marked seasonality and 30 demonstrated an obvious trend decline in mortality.

**Figure 1:**
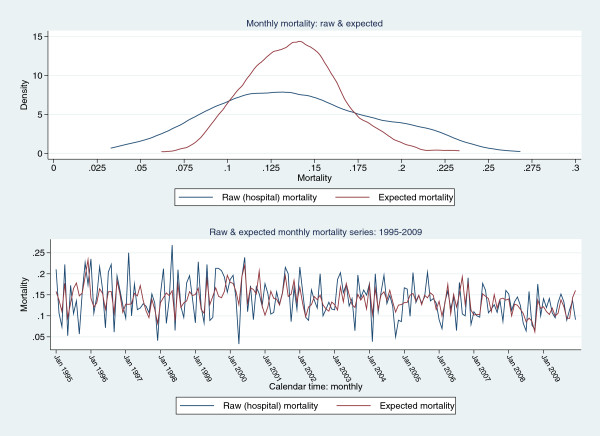
Upper panel: Kernel density estimates of raw and expected mortality. Lower panel: Time series (1995-2009) of mean monthly raw and expected mortality.

**Figure 2:**
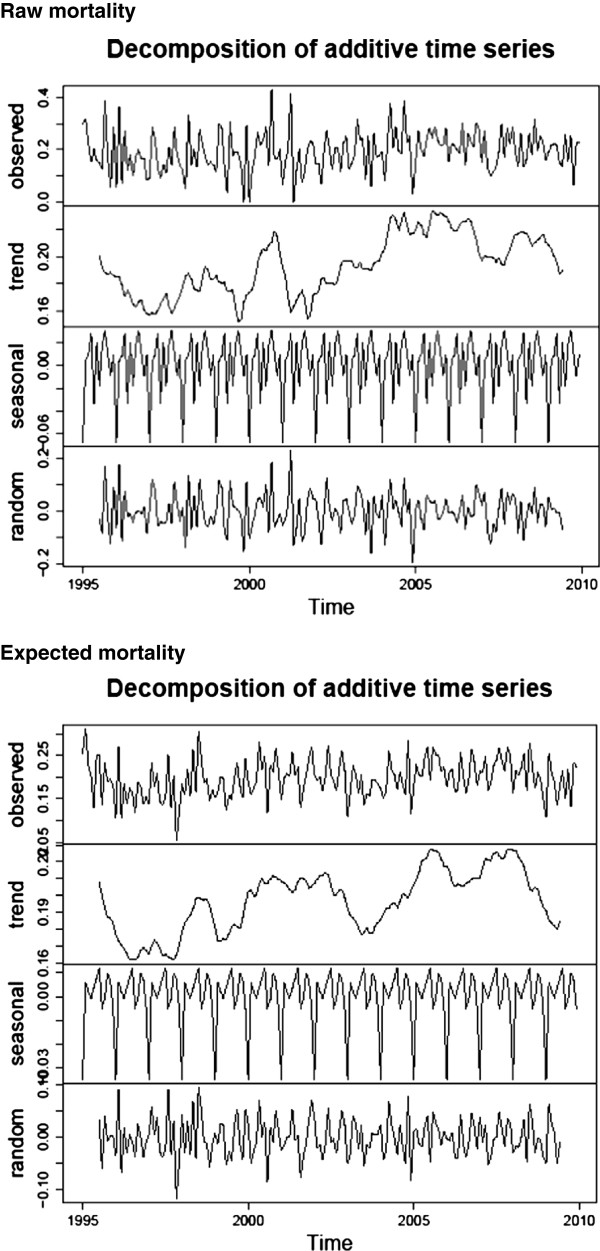
Decomposition plot of raw (upper panel) and expected (lower panel) mortality series.

**Figure 3:**
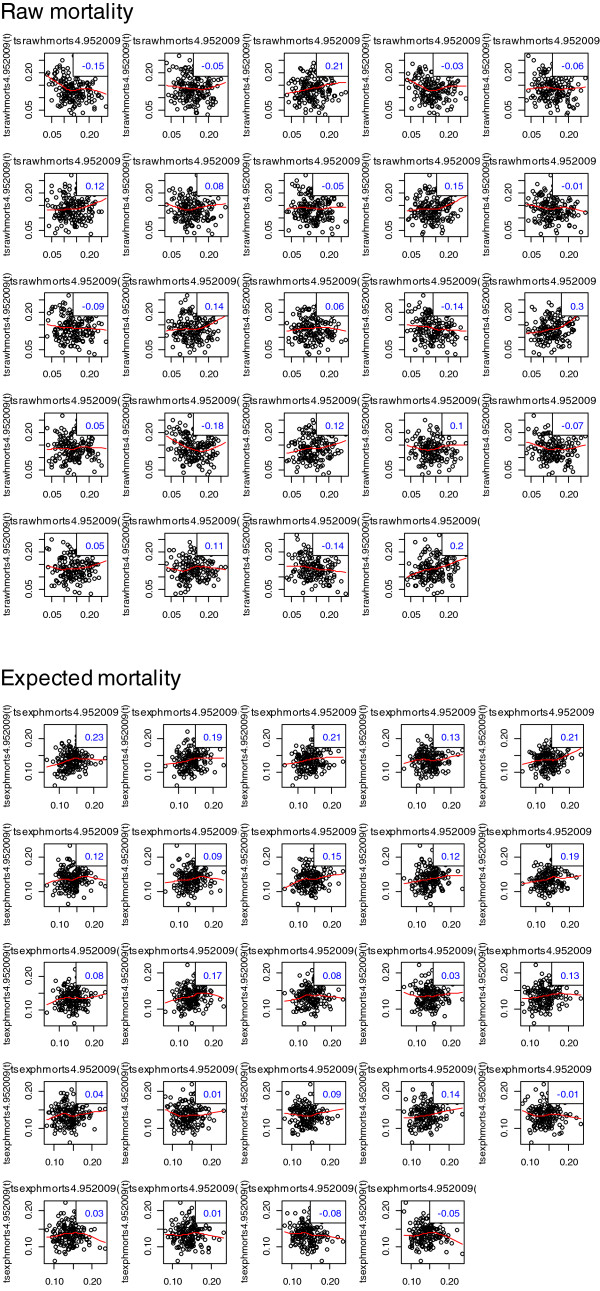
**Lagplot of series versus lagged values (to lag**_**24**_**); upper panel, raw mortality; lower panel, expected mortality.**

Data from site “4” over 1995-2009 was used to generate an exemplar mortality time series. The mean raw mortality was 0.139(0.047) with skewness 0.216 and kurtosis 2.53; and the expected mortality was 0.138(0.028) with skewness 0.361 and kurtosis 3.47. The Shapiro-Wilk normality test was not rejected for either series (P =0.23 for both series). Kernel density estimates of raw and expected mortality are seen in Figure 1 (upper panel), with obvious difference in the degree of kurtosis between the two series. Time series plots, 1995-2009, for raw and expected (RA-)mortality are seen in the lower panel; a gradual time-decline in mortality for both series is evident. Additive seasonal decomposition of both series is seen in Figure 2, revealing marked seasonality and a trend decline in mortality. Autocorrelation plots are seen in Figure 3, showing correlation (positive and negative) decreasing variably with increase in lag in both series.

**Figure 4:**
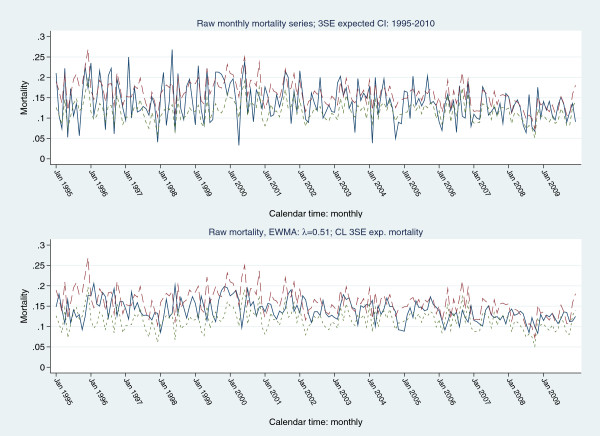
Upper panel: raw mortality series with 3SE control limits of expected mortality; lower panel: EWMA (λ=0.51) of raw mortality series for anticipated 5% increase in raw mortality, 5% fale positive rate and desired ARL=6 months with 3SE control limits of expected mortality.

**Figure 5:**
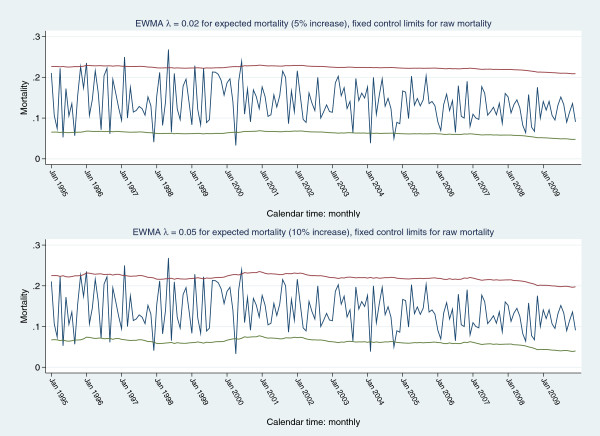
Raw mortality series with EWMA fixed 3SE control limits: upper control limit (red line), lower control limit (green line), signal (navy line); for anticipated 5% (upper panel) and 10% (lower panel) increments in expected mortality.

**Figure 6:**
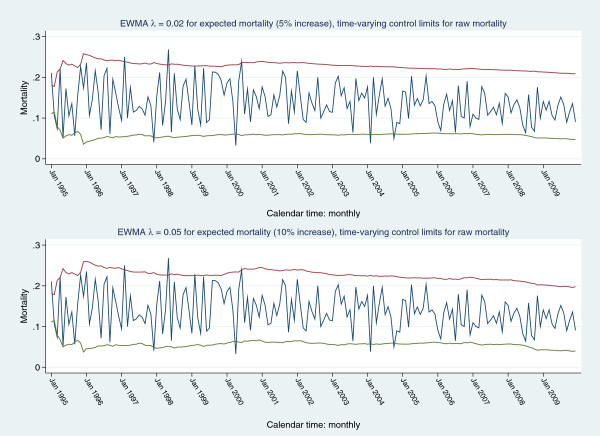
Raw mortality series with EWMA time-varying 3SE control limits: upper control limit (red line), lower control limit (green line), signal (navy line); for anticipated 5% (upper panel) and 10% (lower panel) increments in expected mortality.

Figure 4 displays a plot of raw mortality series with control limits as 3SE of expected mortality (upper panel) and a scenario based mortality increment of 5% (5% false positive rate and desired ARL= 6 months) with control limits as 3SE of expected mortality. Frequent signalling is seen in both panel-plots. Figure 5 shows a plot of the raw mortality series with (fixed) EWMA 3 SE control limits derived from a projected 5% (upper panel) and 10% (lower panel) increment in expected mortality, assuming: an in-control ARL of 370, mean (expected) mortality 0.1381(0.0276) and target mean (expected) mortality of 0.145 (5% increment) and 0.152 (10% increment), for an EWMA λ = 0.02 and 0.05, respectively (calculations preformed in Stagraphics®). For both 5% and 10% projected increments of expected mortality, the raw mortality series signalled frequently, mainly in the early periods. Figure 6 shows the same scenarios with a time-varying variance EWMA control chart; again, there was frequent signalling of the raw mortality series.

The autocorrelation evident in the raw and expected mortality series suggested a formal time series approach to SPC:

(i) Raw mortality: both the DF-GLS and ADF tests (with trend) rejected the null-hypothesis of presence of a unit-root and the series was de-trended using linear regression (raw mortality against time) and the ***residuals*** (also not evidencing a unit-root) of the linear regression model were used for subsequent formal analysis. The de-trended series from the raw mortality displayed seasonality but, not surprisingly, no trend decline (graphics not shown). An initial additive seasonal ARMA model satisfied conventional diagnostic requirements, but displayed ARCH effects. Of the (G)ARCH models assessed, the most parsimonious was a simple [ARCH-lag_1_, GARCH-lag_1_] model (Table 1). Although the individual GARCH term was nominally non-significant, there was a highly significant (P=0.0001) test of joint significance of the ARCH and GARCH parameters. There was no advantage of either *t* or general error distribution in the development of the (G)ARCH models.

(ii) Expected mortality: trend stationarity was demonstrated by rejection of the null-hypothesis of existence of a unit-root by the DF-GLS and ADF tests (with the trend option) and de-trending (linear regression of expected mortality against time) yielded ***residuals*** (also not evidencing a unit-root) for subsequent formal analysis. A simple (multiplicative) seasonal autoregressive model was generated with no evidence of ARCH effects (Table 1). Although an ARMA(1,1) model satisfied model diagnostic tests, the multiplicative seasonal AR model was favoured on clinical grounds.

**Table 1:** Parameters for GARCH (estimated from raw mortality series; de-trended linear model residuals) and ARMA (estimated from the expected mortality series; de-trended linear model residuals) models

**Model**	**Coefficient**	**P**	**UL_95% CI**	**LL_95% CI**
***Raw mortality***				
ARMA				
Autoregressive parameters				
L24	0.174	0.016	0.033	0.316
Moving average parameters				
L1	−0.148	0.021	−0.274	−0.022
L15	0.265	0.000	0.128	0.402
L17	−0.203	0.003	−0.340	−0.067
ARCH				
L1	0.014	0.464	−0.024	0.052
GARCH				
L1	0.996	0.000	0.931	1.061
***Expected mortality***				
ARMA				
Autoregressive parameters				
L1	0.145	0.045	0.003	0.288
ARMA12				
Autoregressive parameters				
L1	0.132	0.075	−0.031	0.277

*L* lag.

**Figure 7:**
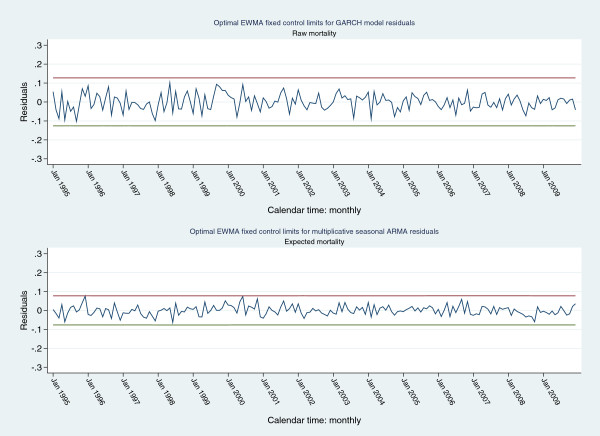
De-trended series (navy line) generating the GARCH (upper panel) and ARMA (lower panel) models with one-step-ahead forecast control limits (3SE); upper control limit (red line), lower control limit (green line).

**Figure 8:**
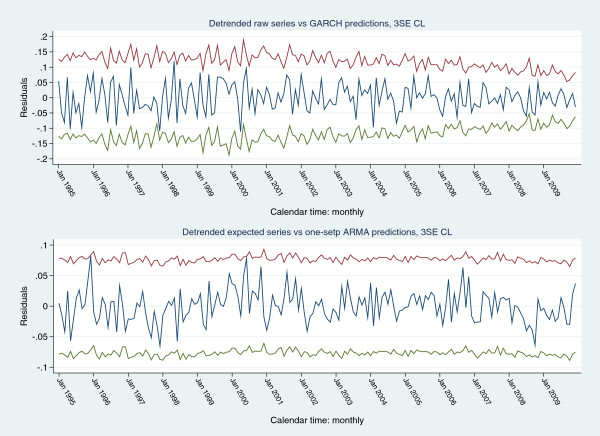
“Optimal” residual-EWMA control chart (3 SE control limits): upper control limit (red line), lower control limit (green line), residuals (navy line); for GARCH (upper panel) and ARMA (lower panel) model residuals, respectively.

**Figure 9:**
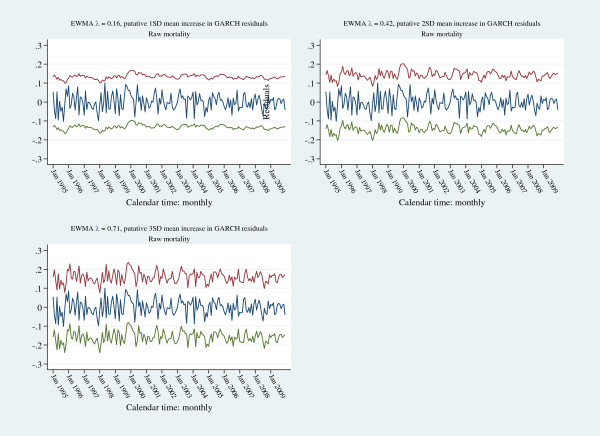
Residual-EWMA control charts (3 SE control limits) for projected 1 (upper left panel), 2 (upper right pane) and 3 (lower left panel) SD increase of residual mean of the GARCH model (for raw mortality series); model residuals (navy line), upper control limit (red line), lower control limit (green line).

Both the GARCH and ARMA models were considered parsimonious and the de-trended signals for each model were within 3SE limits of respective model predictions (Figure 7). The residuals from both the formal GARCH and ARMA models (mean: 0(0.0423) and 0(0.0257) respectively) satisfied multiple criteria of Gaussian white-noise and were within residual-EWMA control limits (default values of “optimal exponential coefficient” in Stata™ statistical software; 3SE control limits; λ = 0.0001 for both series; Figure 8). To address any potential inertial problems consequent upon the small λ, control limits were also established for projected 1 (λ=0.16), 2 (λ=0.42) and 3 (λ=0.71) SD increments of the mean of the GARCH residuals; the latter were within these control limits (Figure 9).

## Discussion

The current analysis of monthly mortality series confirms the existence of autocorrelation and seasonality in both the raw and expected series at the individual ICU level, avoiding any potential confounding at the aggregate level due to Simpson’s paradox. We thus concur with the findings of Alwan [13,50] and Bisgaard and Kulahci [51], who documented the pervasiveness of autocorrelation in a variety of series, industrial and non-industrial. We also established that out-of-control signalling of the raw mortality series with respect to both 3 standard error risk-adjusted and RA-EWMA control limits was not evident with analysis of the residuals from the GARCH time series model. Thus the identification of (G)ARCH processes is an important issue for SPC [35].

As our focus was directed to an understanding of the underlying data-generating process [45] and the performance of the RA-EWMA control limits under conditions of autocorrelation, we deemed it appropriate to also subject the expected series, from which the control limits for the raw mortality series were established, to formal time-series estimation. Not surprisingly, as the underlying mortality estimates from a random coefficient model are obligatorily “smoothed” (see also Figure 1), no ARCH effects, representing “volatility”, were demonstrated and a relatively simple seasonal autoregressive model was established (Table 1). As the EWMA is based upon an ARIMA(0,1,1), that is an integrated moving average process [45,52], it has been applied to autocorrelated data [15], although the majority of studies have used relatively simple non-seasonal autoregressive models (AR(1) or AR(2)) with fixed λ (usually 0.2, which is the default for the SPC model of Statgraphics software). Residual-EWMA charts, in the context of time series modelling, would appear to be more robust than EWMA applied to the original (autocorrelated) data [53,54]. Reynolds and Lu have recommended that under autocorrelation “…traditional control chart methodology should not be applied without modification…” [55] and Human et al. have recently sounded a cautionary note about the robustness of the conventional EWMA [56].

For the classical SPC model, a process is in control if the mean and standard deviation estimate remain within prescribed control limits [57], usually three-sigma; that is, for a normally distributed series, 99.7% of observations should lie within the limits [58] and there a probability of 0.0027 that any point exceeds the control limit [32,59]. However this definition does not necessarily entail the formal time-series notion of stationarity (strict or weak), where the requirement for stationarity is that the first two moments (mean and variance [45,50]) and the autocorrelation function are time-invariant, albeit a stationary processes may be auto-correlated [60]. In the industrial/engineering sphere, practitioner response to process autocorrelation [61] was to embrace a time-series paradigm and apply SPC methods to the residuals of a formal time-series model [45,62], albeit there were different tactical approaches [15,63]; or to develop modified control limit schemes [64-66]. It is instructive to note that the non-model based EWMAST chart (EWMA chart for stationary processes [66]), recommended by Winkel and Zhang [11], pre-supposes a stationary (not “in-control”) process. In a systematic review of the application of statistical process control in healthcare, Thor et al. [5] adduced only one literature reference [67] and a calendar year 2003 monograph which discussed autocorrelation in medical series. As argued by Alwan and Roberts [45], systematic non-random patterns in series make separation of the classic common and special causes difficult, as departures from control, nominally traceable to special causes, are confounded by autocorrelation and, in the current series, seasonality. Two further concerns were raised by the authors; first, the undue emphasis placed upon normality and the (erroneous) assumption that “approximate normality” implies a state of statistical control; and second, in the presence of a well-fitting time series model with residuals consistent with white-noise (“randomness”), it is “…futile to search for departures from statistical control and their corresponding special causes…”. The latter caution resonates with the current finding of frequent signalling of the raw mortality series compared with in-control residuals from an apposite time series model; with respect to the error process, such signalling represents false positivity [13].

Cook and co-workers, “…explicitly compare[d] EWMA(observed) and EWMA(predicted) …[with] thresholds around the EMWA(predicted)…”, employing the EWMA (λ = 0.005-0.020) to “…effectively attenuate noise in the data and smooth an erratic but unbiased risk model” [25], although no criteria of “erratic” were provided. Smoothed control limits for the expected series were also utilised in a review paper by Cook et al. ([68]) and Pilcher et al. ([69], λ = 0.005), albeit the data structure differed; sequential plotting of each patient admission versus monthly mortality rates in the current paper. Our focus and methodology were different, in that we were concerned to both understand and formally model the “noise in the data”. This being said, in the current series, the smoothed EWMA (λ = 0.51) raw series (Figure 4) was demonstrated to signal using 3 standard error expected mortality control limits.

The sophistication of time-series modelling in standard statistical software packages makes the formal analyses of the current study feasible; in particular, automated routines for application of time series models [70]. However, for the application of appropriate SPC to mortality series from multiple ICUs in a data-base, there are unresolved statistical issues [71,72]. From the perspectives of this study, a multivariate approach may be established using more conventional estimators (multivariate GARCH [73] and vector autoregression models [74]) or by newly described hierarchical/functional time series [75,76].

## Conclusions

The underlying data generating process of monthly mortality series at the ICU level displayed autocorrelation and seasonality, with volatility evident in the raw mortality series. Failure to accommodate these characteristics by SPC measures resulted in false-positive signalling. A time series approach to SPC, using residual control charts, would appear to resolve such issues.

## Competing interests

The authors declare that they have no competing interests.

## Authors’ contributions

The study was conceived, designed, (data)-analysed, written and critically revised jointly by both authors (JLM, PJS). Both authors read and approved the final manuscript.

## Pre-publication history

The pre-publication history for this paper can be accessed here:

http://www.biomedcentral.com/1471-2288/13/66/prepub
